# Training Improves Avoidance of Natural Sick Faces: Changes in Visual Attention and Approach Decisions

**DOI:** 10.3390/vision9020039

**Published:** 2025-05-02

**Authors:** Tiffany S. Leung, Krisztina V. Jakobsen, Sarah E. Maylott, Arushi Malik, Shuo Zhang, Elizabeth A. Simpson

**Affiliations:** 1 Department of Psychology, University of Miami, Coral Gables, FL 33124, USA; 2 Department of Psychology, James Madison University, Harrisonburg, VA 22801, USA; 3 Department of Psychiatry and Behavioral Sciences, Duke University, Durham, NC 27708, USA

**Keywords:** visual attention, behavioral immune system, pathogen avoidance, sick face perception

## Abstract

Humans evolved a behavioral immune system to avoid infectious disease, including the ability to detect sickness in faces. However, it is unclear whether the ability to recognize and avoid facial cues of disease is malleable, flexibly calibrated by experience. Thus, we experimentally tested whether we can improve adults’ (*N* = 133) lassitude (sick) face perception, measuring their recognition, avoidance, and visual attention to naturally sick and healthy faces. Participants randomly assigned to a training about disease, but not a control group, were better at avoiding sick people. The disease-trained group also looked more equally between sick and healthy faces when identifying who was sick compared to the control group who looked longer at the sick faces than the healthy faces. Though we detected no group differences in time looking at the eyes and at the mouths, the disease-trained group used these features more to decide who was sick, reflecting key features of the lassitude expression. Our findings suggest that facial sickness perception may be flexible, influenced by experience, and underscore the need for future studies to test how to further strengthen this skill. Ultimately, developing interventions that use this sick face plasticity may reduce disease transmission.

## 1. Introduction

Humans evolved a behavioral immune system that facilitates rapid detection, interpretation, and avoidance of communicable diseases [1]. Part of this system is disease detection, which is theorized to continually calibrate (i.e., flexibly adjust) as people encounter signals of disease [2,3]. A malleable disease detection system with continual calibration would allow individuals to balance avoiding socially transmitted pathogens (risks that fluctuate over time) with the need for engaging in beneficial social interactions.

There is empirical support for a flexible behavioral immune system. Pathogen avoidance may be improved following exposure to disease cues [4,5,6,7,8,9,10,11]. For example, people distance themselves from those who are coughing [12] and the sound of sneezing increases people’s perceived vulnerability to disease [13]. Further, people primed to think about pathogens—either by viewing a video about infectious disease [14], viewing a slideshow of disease-related images (e.g., bacteria on household items) [7], or reading about disease transmission [8]—display more disease vigilance (e.g., avoidance of people with chronic illness).

### 1.1. Sensitivity to Facial Cues of Sickness

One type of disease vigilance that may be flexible is sensitivity to health-related facial cues. For example, watching a video about the dangers of infectious disease increases people’s accuracy in recognizing facial cues of chronic illness [14]. Further, people who read a disease-related story containing disgusting events (e.g., being sneezed on) or view a slideshow with disease-related cues (e.g., people with infected wounds), compared to those in a control condition, have an increased preference for facial qualities associated with healthiness (e.g., attractiveness) [11,15]. Together, these findings suggest that people detect sickness through facial cues and this sensitivity to facial health may be enhanced through priming.

However, it remains unknown whether people can be primed to recognize and avoid facial cues of *contagious* illness, which have important survival consequences. Humans may be especially attuned to visual cues of illness [16]. When people have a contagious illness they may exhibit *lassitude*, a negative emotion characterized by slack facial muscles, drooping eyelids, and slightly parted lips [17]. Indeed, even without priming, humans recognize and automatically avoid sick faces [18,19,20]. However, people’s accuracy in recognizing and avoiding facial sickness is low (<70%) [20] and it is unclear whether this skill is malleable.

### 1.2. Visual Attentional Biases to Sick Faces

Sick faces may receive more attention than other types of stimuli. Evolutionarily relevant social threats, such as angry faces, tend to hold attention more than non-threats [21,22]. In fact, in a categorization task (in which sickness was not task-relevant) event-related potentials reveal that faces edited to appear sick hold attention longer compared to unedited healthy faces [23]. Further, in a dot-probe paradigm, people are slower to shift their attention away from faces with disfigurements, which cue the behavioral immune system, compared to healthy faces, suggesting that faces perceived as unhealthy more strongly hold attention [24,25]. However, it is unclear whether naturally sick faces hold attention longer than healthy faces and whether this attention-holding is flexible.

### 1.3. Current Study

While previous studies demonstrated that people can avoid pathogens using facial cues [19,20], the malleability of this skill—whether sick face perception and its associated visual attention patterns can be enhanced through experiences—is unclear. To improve public health, it may be beneficial to develop methods to support sick face perception, given that even small inaccuracies may have consequences: misjudging a healthy individual as sick may lead to missed social interactions, while failing to recognize sickness may increase the risk of disease transmission [26]. Thus, in the current study, we experimentally tested whether disease training can improve sick face recognition and avoidance while also tracking visual attention to uncover potential mechanisms. We randomly assigned participants to either a brief educational experimental manipulation designed to improve sick face recognition/avoidance (disease training group) or to a control group. We asked adults to identify who they would approach (to capture avoidance) and who they think is sick (recognition) using face photos of people with naturally occurring acute, contagious illnesses and face photos of the same people when healthy. We predicted that the disease training would improve the accuracy of sick face avoidance and recognition and make participants faster at avoiding sick people. We also predicted that sick faces, particularly the eye and mouth regions, would hold visual attention longer than healthy faces, especially for the disease training group. Finally, we predicted the disease training group would be more likely than the control group to report using the eye and mouth regions to identify sickness.

## 2. Materials and Methods

### 2.1. Participants

We recruited adults (*N* = 133) from an undergraduate student research participant pool at the University of Miami (see Table 1 for demographic details). An a priori power analysis in G*Power [27] determined that a sample size of 124 participants would provide 80% power to detect small–moderate effect sizes. The participants received course credit for their participation. The University of Miami Institutional Review Board approved this study.

### 2.2. Materials

#### 2.2.1. Face Stimuli

**Figure 1:**
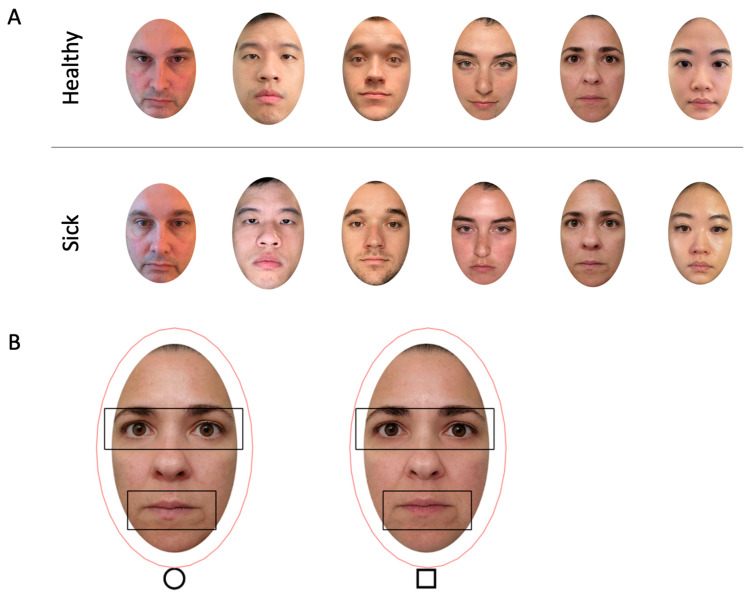
(**A**) Examples of sick and healthy face stimuli. (**B**) Examples of AOIs drawn around the faces (pink ovals), eyes (black rectangles), and mouths (black rectangles).

We included 32 photos from 16 donors of diverse races (5 Asian, 2 Black or African American, 6 White, 1 American Indian/Alaska Native and White, and 2 Black or African American and White), ethnicities (6 Hispanic or Latino and 10 non-Hispanic or Latino), genders (6 men, 2 boys, 7 women, and 1 nonbinary adult), and ages (*M* = 29 years old, *SD* = 17, range: 8–79 years). Each donor contributed two photos: one sick and one healthy (Figure 1, Table S1). All the photos were validated on perceived health in separate samples [19,20]. At the time of the sick photo, the donors reported having a contagious disease, including COVID-19, streptococcal pharyngitis, rhinovirus, and influenza. A within-subjects comparison of sick and healthy faces controlled for between-subjects variables (e.g., facial symmetry or age). The photo donors maintained a neutral facial expression and were instructed to relax their faces (i.e., reduce tension in their facial muscles).

#### 2.2.2. Avoidance Task Materials

The faces were displayed side-by-side with one image of the donor’s face when they were sick and a second image of that same donor’s face when they were healthy. We horizontally and vertically centered each face on its respective left or right side of the image. The faces were displayed on a Dell P2214H Monitor and were sized 4.30–5.20 × 5.90–8.10 cm.

#### 2.2.3. Recognition Task Materials

We again presented the side-by-side face pairs (described above), slightly larger to fit the screen: each face was 299–360 pixels wide (*M* = 327.56, *SD* = 19.67) × 426–581 pixels tall (*M* = 495.06, *SD* = 39.29, which is 7.91–9.52 × 11.27–15.37 cm and 7.54–9.07 × 10.73–14.60°). The faces were spatially separated by 282–340 pixels (*M* = 310.38, *SD* = 19.53), which is 7.46–9.00 cm and 7.11–8.58°. A circle or a square was below each face, allowing the participants to say the shape that corresponded to the face they wanted to select.

We used a Tobii TX300 eye-tracker (Tobii Technology, Danderyd, Sweden), with a remote 58.4 cm monitor (51 cm in width × 28 cm in height) with integrated dark pupil eye-tracking technology, with a resolution of 1280 × 720 pixels, and a sampling rate of 300 hertz. The test room had no windows and a constant illumination of approximately 202 lux. We used the Tobii Studio software version 2.0 (Tobii Technology, Danderyd, Sweden) to collect and summarize the eye-tracking data.

### 2.3. Procedure

The participants were tested in a controlled laboratory setting. Following informed consent, the participants completed demographic questions (e.g., age and race).

#### 2.3.1. Sick Face Avoidance Pre-Manipulation Baseline

**Figure 2:**
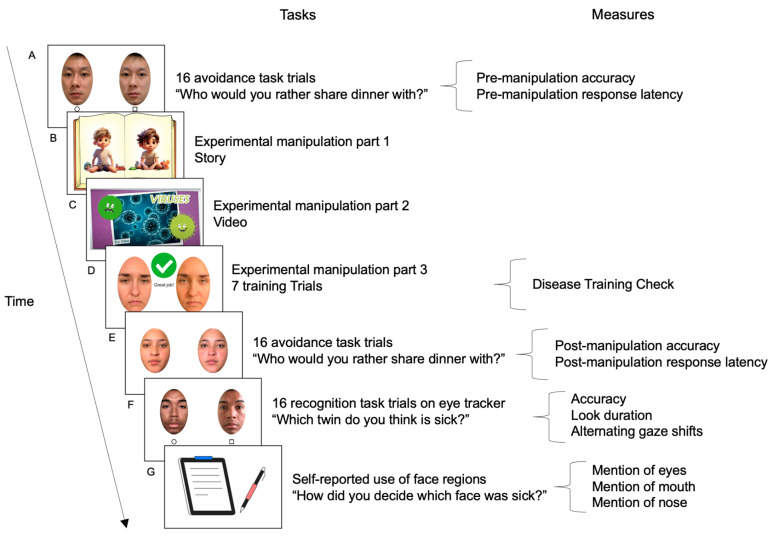
Study procedure tasks and measures: (**A**) pre-manipulation baseline avoidance task, (**B**) experimental manipulation: interactive story, (**C**) experimental manipulation: video, (**D**) interactive experimental manipulation: training trials, (**E**) post-manipulation avoidance task, (**F**) recognition task with eye tracking, and (**G**) participant self-report of what facial features they used to identify sick faces.

To verify that the participants did not differ in their sick face perception prior to the experimental manipulation, we collected a baseline measure of sickness avoidance (Figure 2A). We chose this task that did not explicitly mention sickness to ensure that we would not unintentionally prime participants (the control group, in particular) to think about sickness. The participants were seated in front of a computer screen on which they viewed side-by-side pairs of sick and healthy faces that were described as “twins” to increase the credibility of the scenario, though they were in fact from the same person [20]. The participants were instructed to imagine they are in a restaurant where there are only two seats available. As each available seat is next to a twin, they must decide which twin they would prefer to sit next to and share dinner with. The participants completed 2 practice trials with cartoon characters. After that, the participants viewed the experimental trials, each consisting of two human faces side-by-side. The experimenter asked, “Who would you rather share dinner with?” and the participants responded with a mouse click. The participants completed 16 trials randomized using a Latin Square Design. The side of the sick face was counterbalanced to ensure it was equally often on the left and the right. Each face pair remained on the screen until the participant made a choice.

#### 2.3.2. Disease Training Experimental Manipulation

We included a three-part experimental manipulation—a story (part 1), a video (part 2), and a training session (part 3)—based on previous studies that had success with providing information about disease transmission and videos related to infectious disease [8,14]. After random assignment to either the disease training condition or the control condition, the participants engaged in an interactive 3-minute story with an experimenter (experimental manipulation part 1; Figure 2B). For the disease training condition, the story described facial cues of sickness and the importance of disease prevention behaviors (e.g., maintaining a safe distance from sick people) and included images of sick and healthy faces, generated by Midjourney (Version 5.2) artificial intelligence, to illustrate how disease spreads and how temporary social distancing can reduce illness transmission. The participants in the control condition heard a story about animals while viewing Midjourney-generated images of characters and animals. The control condition story was unrelated to disease to prevent priming the participants. The stories were matched on length, complexity, and interactive components and featured the same characters. Both stories were read aloud by the experimenter and included audio clips (i.e., a person coughing in the disease training and a parrot in the control condition) and open-ended questions (e.g., “What would you think if you heard that sound inside this room?”). We verified that the participants were attentive by asking them to recall where the story took place.

Following the story, the participants in the disease training condition watched an animated 3-minute video (experimental manipulation part 2; Figure 2C) about infectious diseases (e.g., influenza) that included descriptions of virus transmission, immune system responses, and symptoms [28]. The participants in the control condition watched a 3-minute video about birds that was unrelated to disease [29]. The two videos were matched in length and featured the same adult woman presenter, who did not appear elsewhere in the study.

In the final portion of the experimental manipulation, the participants completed a training session (experimental manipulation part 3; Figure 2D): in the disease training condition, the participants viewed examples of sick and healthy faces and were told, “When someone is feeling sick and their body is fighting off germs, their face may look different. Faces can give us clues about how someone is feeling, which helps us figure out if they are sick or healthy”. The participants were asked to note some of the commonalities among the sick faces. The experimenter then confirmed the participants’ observations and, if necessary, provided further details about facial features common in sick faces paired with photo examples. The experimenter explained that people tend to have drooping eyes [18] and relaxed facial muscles [17,30] when they are sick. The participants then completed 7 training trials of sickness recognition with feedback on their choices. If the participant chose the incorrect face, they were reminded of facial features associated with sickness (e.g., drooping eyes). The participants in the control condition completed a parallel bird recognition training that focused on the color and body features of cockatiels and parakeets. We asked the participants to identify features of cockatiels, and explained, “When you see a cockatiel, you might notice that the hair sticks up. This is called a crest, which sometimes sticks up when it’s feeling excited or happy”. The participants then completed 7 training trials of bird recognition with feedback on their choices. In total, the experimental manipulation lasted approximately 8 min.

#### 2.3.3. Sick Face Avoidance Post-Manipulation

Following the experimental manipulation, the participants repeated the pre-manipulation avoidance task (Figure 2E). The tasks were identical prior to the manipulation and following the manipulation.

#### 2.3.4. Sick Face Recognition (Eye Tracking)

Within the same testing room, the participants moved to the eye-tracking screen and were seated approximately 60 cm from the screen. Following a 9-point calibration, we tracked the participants’ gaze while they viewed faces. We asked the participants to imagine that they are doctors working in a hospital with the goal of identifying which individual in a pair of twins is sick (Figure 2F). As with the avoidance tasks, the face pairs were presented side-by-side and were described as twins but were in fact from the same donor. The participants completed 2 practice trials with cartoon characters and then viewed 16 test trials consisting of side-by-side sick-healthy face pairs. The experimenter asked, “Which twin do you think is sick?” and the participants verbally reported their choices by saying the shape that corresponded to the face they wanted to choose (i.e., circle or square). The experimenter, blind to which faces were sick and healthy, recorded the participants’ responses to move to the next trial. The side on which the sick face appeared was counterbalanced to ensure it was equally often on the left and the right, and the trials were randomized using a Latin Square Design. After the task, we asked the participants, “How did you determine which one of the faces was sick?”

### 2.4. Measures

#### 2.4.1. Sickness Avoidance and Recognition Accuracy Scores

We calculated the number of correct trials in the pre-manipulation baseline avoidance task, post-manipulation avoidance task, and recognition task.

#### 2.4.2. Sickness Avoidance Speed—Manual Response Latency

For the avoidance task, we extracted the duration of time from when the trial first appeared to when the participant made their decision about which face to approach using a mouse click.

#### 2.4.3. Visual Attention Holding to Sickness—Look Duration Difference Scores

Using look duration as a measure of visual attention allows for the capture of subtle and covert sick face processing skills, which may be more robust compared to other behavioral measures, such as self-report responses that primarily capture overt and conscious awareness of disease cues [19]. For the recognition task, we calculated a mean look duration difference score for each participant by subtracting the total time looking at healthy face AOIs from the total time looking at sick face AOIs, capturing differences in attention holding [19]. Thus, positive difference scores in the recognition task indicated that the participants looked longer at the sick faces than the healthy faces, suggesting that sick faces hold attention, and negative difference scores indicated longer looking at the healthy faces than the sick faces, suggesting that healthy faces hold attention. We also calculated a difference score for looking at the eye regions, subtracting the total time looking at the healthy face eye AOIs from the total time looking at the sick face eye AOIs. Similarly, we calculated a difference score for looking at the mouth region AOIs, subtracting the time looking at the healthy face mouth from the time looking at the sick face mouth AOIs. We also did this for the remaining face regions (e.g., nose and cheeks): we calculated a difference score for looking at the non-eye–mouth regions (face AOI minus the eye and mouth AOIs), subtracting the time looking at the healthy face non-eye–mouth regions from the time looking at the sick face non-eye–mouth regions.

#### 2.4.4. Visual Comparison of Sickness—Number of Alternating Gaze Shifts

We extracted the number of visits to each face AOI, with each visit defined as one or more consecutive fixations within an AOI prior to a fixation outside the AOI. To index the number of times the participants looked back and forth between the sick and healthy faces, reflecting comparisons between the two images [31], we summed the number of visits to the sick face AOI and the number of visits to the healthy face AOI and subtracted one (to account for the fact that they had to make an initial look). We calculated similar alternating gaze scores for looking back and forth at the eye regions (eye AOIs) between the faces and at the mouth regions (mouth AOIs) between the faces.

#### 2.4.5. Report of Facial Sickness Cues

We transcribed the participants’ verbal responses to the question, “How did you determine which one of the faces was sick?” We then searched for the most common themes reported and coded whether each participant mentioned them or not (yes/no).

### 2.5. Analytic Approach

All analyses were conducted in R and R Studio Version 2023.09.1.

#### 2.5.1. Preliminary Analyses

We conducted an independent samples *t* test to confirm that the participants in the disease training group did not differ from the control group in their baseline sickness avoidance accuracy (number of correct trials) prior to the experimental manipulation.

We conducted one-sample *t* tests to confirm that participants’ accuracy at avoiding and recognizing sick faces was above chance performance (0.50) overall, as previously reported in adults [20].

#### 2.5.2. Primary Analysis 1 (Prediction 1) Sickness Avoidance: Accuracy and Speed Before and After Disease Training

We conducted two 2 (Condition: disease training, control) × 2 (Time: pre-manipulation, post-manipulation) mixed-design ANOVAs to compare accuracy (number of correct trials) and manual response latency in the avoidance task.

#### 2.5.3. Primary Analysis 2 (Prediction 2): Sickness Recognition: Accuracy and Visual Attention

We also conducted independent samples *t* tests (or Welch’s *t* tests, when group variances were significantly unequal) to compare the disease training group and the control group on three types of measures collected in the recognition task: (1) accuracy (number of correct trials), (2) look duration difference scores (i.e., sick minus healthy) to the face eyes, and mouth, and (3) the number of alternating gaze shifts to the face, eyes, and mouth.

#### 2.5.4. Primary Analysis 3 (Prediction 3): Facial Sickness Cues

Finally, we conducted three logistic regressions to test whether the disease training group was more likely than the control group to report using the eye, mouth, and nose regions to identify the sick face.

## 3. Results

### 3.1. Preliminary Results

#### 3.1.1. Data Inclusion

We excluded the participants who failed the attention check and removed a small number of cases across specific measures that were outliers (see Supplementary Materials for details).

#### 3.1.2. Baseline (Pre-Experimental Manipulation) Check

We confirmed that participants in the disease training group (*M* = 10.44 correct trials [65% accuracy], *SD* = 2.01) did not differ from the control group (*M* = 10.39 correct trials [65% accuracy], *SD* = 1.43) in their baseline sickness avoidance accuracy (number of correct trials) in the pre-manipulation baseline, *t*(108.15) = 0.18, *p* = 0.861.

#### 3.1.3. Experimental Manipulation Disease Training Check

We first checked the disease training group’s accuracy in the seven training trials (experimental manipulation part 3), confirming that they performed above chance in their sickness recognition (*M* = 6.5 correct trials, *SD* = 0.59, range: 5–7), *t*(65) = 41.43, *p* < 0.001, *d* = 4.93. Given that previous studies reported relatively low accuracy (below 70% accuracy) on this task [20], 93% accuracy in the current training task suggests that the disease training was effective.

#### 3.1.4. Post-Experimental Manipulation Avoidance Replication Check

We replicated previous findings that, pooling all the participants across both groups, the participants were above chance (eight trials; 50% accuracy) at avoiding sick faces with a one-sample *t* test (*M* = 10.41 correct trials [65% accuracy], *SD* = 1.74, range: 6 to 15), *t*(122) = 252.83, *p* < 0.001, *d* = 1.39. We also replicated these findings within each group: the disease training group (*M* = 10.39 correct trials [65% accuracy], *SD* = 1.43, range: 6–15), *t*(60) = 153.5, *p* < 0.001, *d* = 1.21 and the control group (*M* = 10.44 correct trials [65% accuracy], *SD* = 2.01, range: 6–13), *t*(61) = 218.15, *p* < 0.001, *d* = 1.67, were both above chance in their avoidance of sick faces, consistent with a prior study using these tasks [20].

### 3.2. Sickness Avoidance: Accuracy and Speed Before and After Disease Training (Prediction 1)

#### 3.2.1. Sickness Avoidance Accuracy

Our ANOVA on sickness avoidance accuracy (number of correct trials) in the avoidance task revealed marginal main effects of condition, *F*(1, 120) = 3.08, *p* = 0.082, η^2^ₚ = 0.02, in which the disease training group (*M* = 10.80, *SD* = 1.76) was marginally more accurate than the control group (*M* = 10.39, *SD* = 1.64). We also detected a marginal main effect of time, *F*(1, 120) = 3.41, *p* = 0.067, η^2^ₚ = 0.03, in which sickness avoidance accuracy in the post-manipulation task (*M* = 10.77, *SD* = 1.68) was marginally greater than in the pre-manipulation task (*M* = 10.41, *SD* = 1.74), potentially because both groups were slightly improving with practice as the tasks progressed, even without feedback.

**Figure 3:**
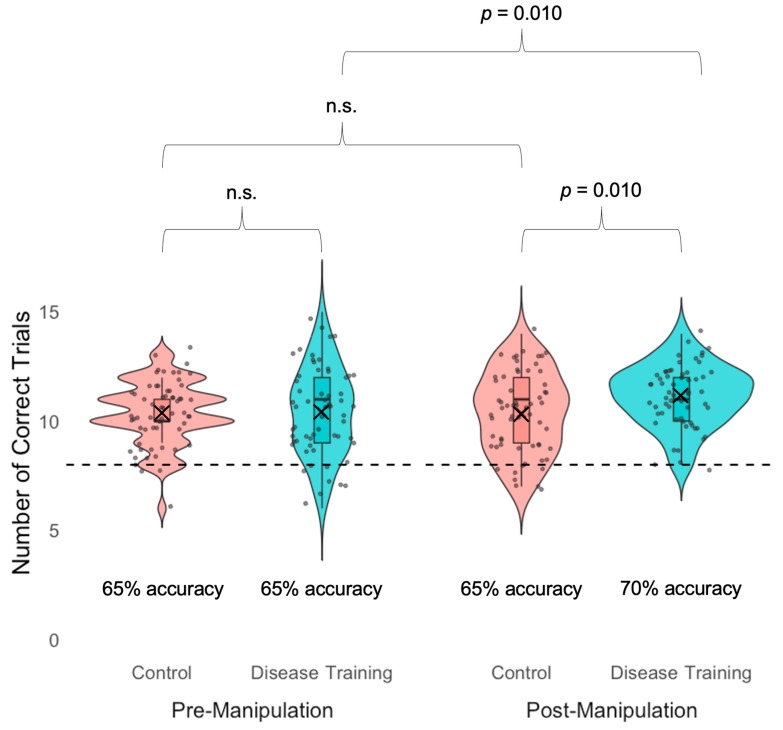
Accuracy in the avoidance task (“Who would you rather share dinner with?”) measured by the number of correct trials (out of 16 total) before the experimental manipulation (pre-manipulation; left) and after the experimental manipulation (post-manipulation; right) for the control condition group (red) and the disease training group (blue). Boxes indicate the first and third quartiles, whiskers indicate 1.5× above and below the interquartile range, horizontal lines within the boxes indicate medians, Xs indicate means, and n.s. indicates not statistically significant (*p*s > 0.10). All the subgroups were above chance (dashed line; 8 out of 16 trials; 50% correct).

These main effects were qualified by a marginal interaction between condition and time, *F*(1, 120) = 3.47, *p* = 0.065, η^2^ₚ = 0.03. As reported above, in the pre-manipulation avoidance task (baseline check), the disease training group (*M* = 10.44 correct trials [65% accuracy], *SD* = 2.01) did not differ from the control group (*M* = 10.39 correct trials [65% accuracy], *SD* = 1.43), *t*(108.15) = 0.18, *p* = 0.861. However, as predicted, in the post-manipulation avoidance task, the disease training group (*M* = 11.16 correct trials [70% accuracy], *SD* = 1.39) was more accurate (greater number of correct trials) than the control condition group (*M* = 10.38 correct trials [65% accuracy], *SD* = 1.85), *t*(113.3) = 2.63, *p* = 0.010, *d* = 0.48. As predicted, the disease training group improved from before to after the manipulation (from 10.44 trials correct [65% accuracy] to 11.16 trials correct [70% accuracy]), *t*(60) = 2.68, *p* = 0.010, *d* = 1.16, while the control group showed no changes (pre-manipulation: *M* = 10.40, *SD* = 1.43; post-manipulation: *M* = 10.39, *SD* = 1.85), *t*(61) < 0.01, *p* > 0.99, (Figure 3).

#### 3.2.2. Sickness Avoidance Speed

Our ANOVA on sickness avoidance manual response latency revealed a main effect of condition, *F*(1, 126) = 25.83, *p* = 0.004, η^2^ₚ = 0.06, in which the disease training group (*M* = 3.82, *SD* = 1.51) responded more slowly, taking more time to make their decisions, than the control group (*M* = 1.51, *SD* = 1.22). We also found a main effect of time, in which the participants responded more slowly in the pre-manipulation task (*M* = 3.73, *SD* = 1.38) than in the post-manipulation task (*M* = 3.28, *SD* = 1.41), *F*(1, 125) = 13.40, *p* < 0.001, *d* = 0.18, even without feedback. The participants, overall, grew slower and more accurate as the study progressed, possibly because simply by performing the avoidance task, participants’ face perception skills were improving.

**Figure 4:**
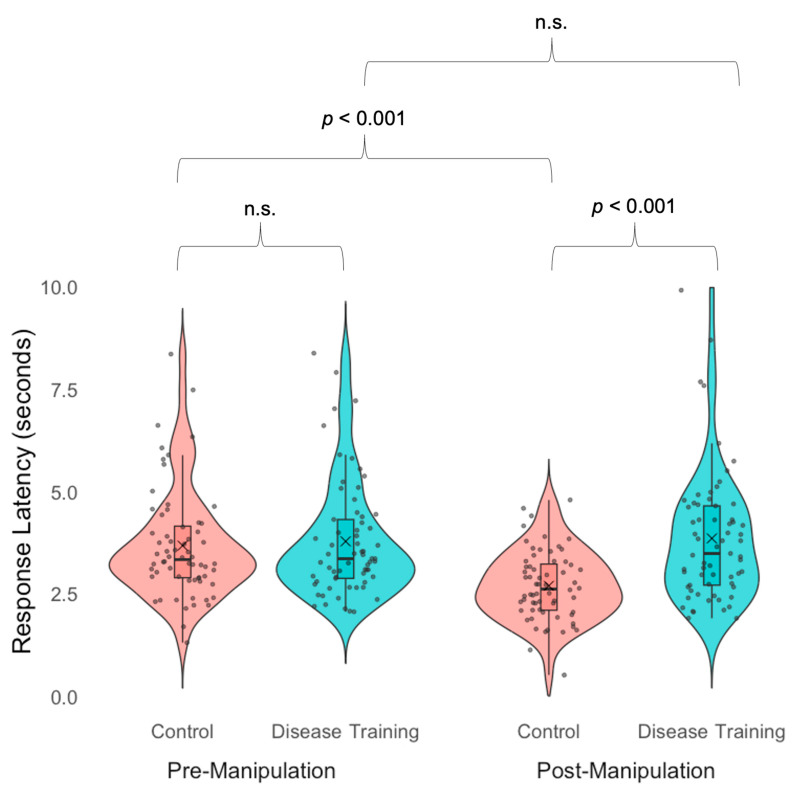
Manual response latency (seconds) in the avoidance task (“Who would you rather share dinner with?”) before the experimental manipulation (pre-manipulation; left) and after the experimental manipulation (post-manipulation; right) for the control condition group (red) and the disease training group (blue). Boxes indicate the first and third quartiles, whiskers indicate 1.5× above and below the interquartile range, horizontal lines within the boxes indicate medians, Xs indicate means, and n.s. indicates not statistically significant (*p*s > 0.10).

These main effects were qualified by an interaction between condition and time, *F*(1, 125) = 18.52, *p* < 0.001, η^2^ₚ = 0.23, Figure 4. In the pre-manipulation avoidance task, we detected no difference in manual response latency between the disease training group (*M* = 3.78, *SD* = 1.42) and the control group (*M* = 3.67, *SD* = 1.35), *t*(127) = 0.43, *p* = 0.669, potentially because both groups carefully examined both faces before making their decision. However, in the post-manipulation avoidance task, the disease training group (*M* = 3.85, *SD* = 1.60) was slower than the control condition group (*M* = 2.69, *SD* = 0.85), *t*(127) = 5.13, *p* < 0.001, *d* = 0.90. This finding may indicate a speed–accuracy trade-off in which the disease training group was slower to respond but more accurate in their responses compared to the control group. The disease training group showed no changes in their sickness avoidance manual response latency from the pre- to post-manipulation task, *t*(64) = 0.56, *p* = 0.572, while the control group had slower responses in the pre- than the post-manipulation task, *t*(62) = 8.15, *p* < 0.001, *d* = 1.96 (Figure 4). The disease training group may have taken more time to make their choices in the post-manipulation task, because they received new task-relevant information in the disease training, altering their interpretation of the task instructions (i.e., they used health cues even though not explicitly instructed to). In contrast, for the control group, the task remained the same, so they showed a practice effect.

### 3.3. Sickness Recognition: Accuracy, Visual Attention, and Report of Facial Sickness Cues (Prediction 2)

#### 3.3.1. Sickness Recognition Accuracy

We did not detect a statistically significant difference between the disease training group (*M* = 11.15, *SD* = 1.85) and the control condition group (*M* = 11.37, *SD* = 1.84) for the number of correct trials in the post-manipulation sickness recognition task, *t*(127) = 0.70, *p* = 0.485. These findings indicate that our disease training did not seem to improve post-manipulation explicit sickness recognition accuracy, though we did not have a pre-manipulation task for this measure.

#### 3.3.2. Look Duration to Faces and Face Regions

**Figure 5:**
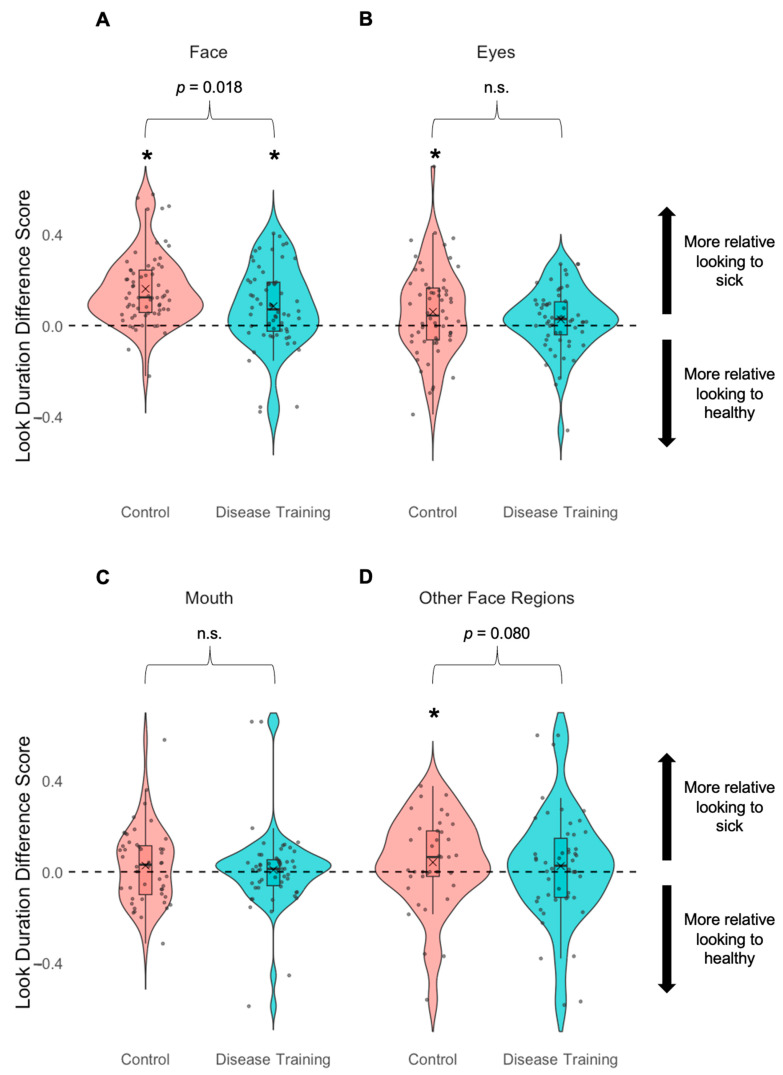
Look duration difference scores in the recognition task (“Which twin do you think is sick?”) after the experimental manipulation (post-manipulation only) for the control condition group (red) and the disease training group (blue), reflecting visual attention to the (**A**) faces, (**B**) eye regions, (**C**) mouth regions, and (**D**) remaining face regions. The number of seconds looking at the healthy face/eyes was subtracted from the number of seconds looking at the sick face/eyes, so scores closer to zero (dashed line) indicate more equal looking at sick and healthy faces, and larger absolute value scores indicate more differential looking, with positive values indicating more looking at the healthy faces and negative values indicating more looking at the sick faces. Boxes indicate the first and third quartiles, whiskers indicate 1.5× above and below the interquartile range, horizontal lines within the boxes indicate medians, Xs indicate means, and n.s. indicates not statistically significant (*p*s > 0.10). One-sample *t* tests indicated looking was different from chance (dashed line), * *p*s < 0.05.

However, the groups differed in their post-manipulation look duration at the faces (i.e., difference scores: number of seconds looking at the healthy face subtracted from the number of seconds looking at the sick face): The control group (*M* = 0.17, *SD* = 0.16) had larger difference scores than the disease training group (*M* = 0.10, *SD* = 0.17), *t*(133) = 2.40, *p* = 0.018, *d* = 0.46 (Figure 5). This finding suggests that the disease training group looked more equally at sick and healthy faces (*M* = 1.25 s, *SD* = 0.73 s, and *M* = 1.17 s, *SD* = 0.79 s, respectively), while the control group looked more to the sick faces (*M* = 1.18 s, *SD* = 0.45 s) relative to the healthy faces (*M* = 1.02 s, *SD* = 0.39 s). These findings suggest that the disease training altered the participants’ relative viewing times to sick and healthy faces.

For the post-manipulation look duration difference scores for the participants’ attention to the eyes (look duration to healthy eyes subtracted from look duration to sick eyes), we detected no difference between the disease training group (*M* = 0.03, *SD* = 0.14) and the control group (*M* = 0.06, *SD* = 0.20), *t*(105.77) = 1.08, *p* = 0.281. However, while the control group’s eye difference score was above chance, *t*(59) = 2.39, *p* = 0.020, *d* = 0.31, the disease training group’s difference score was at chance, *t*(57) = 1.49, *p* = 0.142. These findings suggest that the control group was looking more at the sick face eyes than at the healthy face eyes, but the disease training group was looking more equally at the sick and healthy face eyes.

For the post-manipulation look duration difference scores for their attention to the mouth (look duration to healthy mouth subtracted from look duration to sick mouth), we detected no difference between the disease training group (*M* = 0.01, *SD* = 0.18) and the control group (*M* = 0.03, *SD* = 0.17), *t*(100) = 0.54, *p* = 0.591. Neither group’s mouth difference score was above chance (disease training group: *t*(53) = 0.35, *p* = 0.731; control group: *t*(47) = 1.14, *p* = 0.261), indicating both groups looked for similar amounts of time to the sick and healthy face mouth regions.

For the look duration to the remaining face parts (face AOI minus eye and mouth region AOIs) in the post-manipulation sickness recognition task, the disease training group’s difference scores did not differ from zero (*M* = 0.00, *SD* = 0.31, *t*(50) = 0.08, *p* = 0.937), indicating they looked about equally between the sick and healthy faces’ remaining face parts. The disease training group’s difference scores were less than those of the control group (*M* = 0.12, *SD* = 0.36), *t*(89) = 1.77, *p* = 0.080, *d* = 0.37, who spent more time looking at the sick faces’ remaining parts relative to the healthy faces’, and did so at rates above chance, *t*(39) = 2.15, *p* = 0.038, *d* = 0.34. These findings mirror the other effects of the disease training group looking more equally at the sick and healthy faces (entire face AOIs), eyes, and mouths, while the control group consistently appears to show greater looking to all parts of the sick faces relative to the healthy faces.

#### 3.3.3. Gaze Alternations

**Figure 6:**
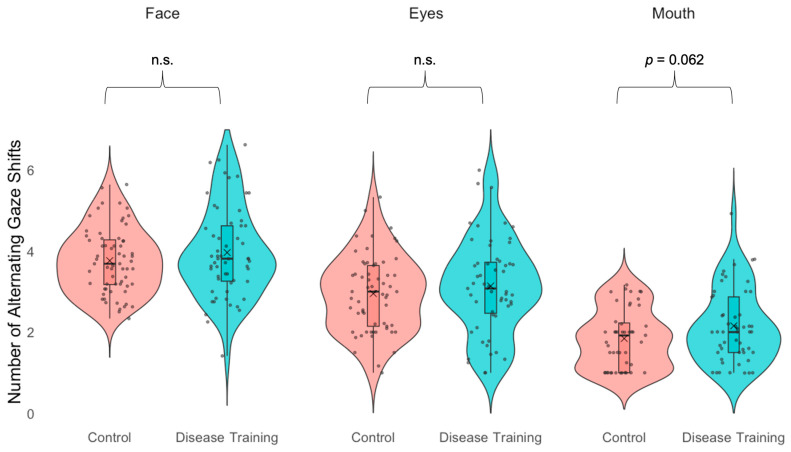
Alternating gaze shifts back and forth between the sick and healthy faces (left), eye regions (middle), and mouth regions (right) in the recognition task (“Which twin do you think is sick?”) for the control condition group (red) and the disease training group (blue). Boxes indicate the first and third quartiles, whiskers indicate 1.5× above and below the interquartile range, horizontal lines within the boxes indicate medians, Xs indicate means, and n.s. indicates not statistically significant (*p*s > 0.10).

Additionally, the disease training group (*M* = 4.19, *SD* = 1.71) did not differ from the control group (*M* = 3.89, *SD* = 1.11) in the number of alternating gaze shifts between sick and healthy faces, *t*(126) = 1.18, *p* = 0.241, Figure 6. The disease training group (*M* = 3.14, *SD* = 1.17) also did not differ from the control group (*M* = 2.95, *SD* = 0.94) in the number of alternating gaze shifts between the sick and healthy faces’ eyes, *t*(114) = 0.94, *p* = 0.348. However, the disease training group (*M* = 2.15, *SD* = 0.91) had marginally more alternating gaze shifts between the sick and healthy faces’ mouths than the control group (*M* = 1.84, *SD* = 0.72), *t*(97) = 1.89, *p* = 0.062, *d* = 0.38. This finding is consistent with what we predicted, given that drooping corners of the mouth is a crucial piece of the lassitude expression (Schrock et al., 2020) [17].

#### 3.3.4. Report of Facial Sickness Cues (Prediction 3)

Finally, a logistic regression revealed that the participants in the disease training group (92%) were more likely than those in the control group (66%) to report using the eyes to determine which person was sick, *b* = 1.81, *SE* = 0.53, *z* = 3.40, *p* < 0.001, 95% CI: 0.84, 2.97; the disease training group was 6.14 times more likely to say eyes compared to the control group. See Table S2 for representative examples of participant responses. The participants in the disease training group (40%) were also more likely than those in the control group (18%) to report using the mouth or lips to determine which person was sick, *b* = 1.08, *SE* = 0.41, *z* = 2.65, *p* = 0.008, 95% CI: 0.30, 1.91. These results are in line with the visual attention differences between the disease training and control groups in their attention to these face regions. A limited number of the participants in the disease training group (0%) and the control group (5%) reported using the nose. See Table 2 for a summary of the primary results.

## 4. Discussion

Given that contagious diseases can spread through brief social interactions, it is important to understand how to support the behavioral immune system. Yet, it is unclear whether the sick face detection system is malleable. Building on previous studies reporting people differentiate between sick and healthy faces [19,20], the current study is the first, to our knowledge, to test whether disease training improves the avoidance and recognition of naturally sick faces with acontagious illness. The participants who were primed to think about pathogens and trained on facial cues of sickness were more accurate at avoiding sick people and displayed differing attentional patterns on faces when evaluating health compared to the control group. Our study reveals that there is potential for improvement in sickness perception for faces with confirmed contagious diseases, similar to previous studies reporting that it is possible to increase vigilance towards potentially unhealthy individuals (e.g., people with physical deformities and older people [4,32,33]. Faces are more consistently available than other cues (e.g., body odor and biological motion) and are detected and processed more rapidly than nearly any other stimulus [34,35,36]. Thus, improving sensitivity and visual attention to facial cues of natural, contagious illness may be beneficial for protecting health [18].

Given these potential benefits, we trained the participants on specific facial cues of illness (e.g., drooping eyelids and downturned corners of the mouth). Those who were trained were more likely to report using these facial regions, which co-occurred with both increased accuracy of sick face avoidance and changes in visual attention to sick and healthy faces. We used faces of people who were naturally sick, paired with the same peoples’ faces when they were healthy, enabling us to control various individual differences (e.g., age, weight, race, and attractiveness). Overall, our results suggest that avoidance of sick faces can be improved with training, consistent with the proposal of a flexible behavioral immune system.

### 4.1. Disease Training Improves Sickness Avoidance (Prediction 1)

The disease training group was slower and more accurate than the control group in deciding who to approach. In the post-manipulation task, the disease training group, compared to the control group, took more time before making their responses, potentially reflecting greater thoughtfulness and careful examination of the facial features associated with health. Of note, while there is typically a speed and accuracy trade-off [37], we found that the disease training group improved accuracy but not at the expense of speed (i.e., there was no change in response latency from pre- to post-manipulation). Our findings suggest that disease training supports both accurate and efficient responses.

Our goal of using the avoidance task was to simulate a common social situation that people may encounter when making decisions about how to spatially navigate around others (e.g., waiting rooms and public transport). However, given that we removed all contextual information (e.g., movement, voice, posture, and odor) that people have in the real world, the fact that we still found accuracy improvement in the disease-trained group is particularly notable. Nonetheless, we acknowledge that the observed effect sizes were small; therefore, future studies are needed to enhance this type of disease training by lengthening the training to include additional practice trials to improve generalizability across a broader range of faces and by tracking how visual attention changes with learning about sick faces.

### 4.2. Disease Training Alters Visual Attention During Sickness Recognition (Predictions 2 and 3)

The participants in the disease training group and the control group did not differ in their accuracy of explicitly recognizing sick people. However, their attention patterns to the faces did differ, suggesting that the experimental manipulation altered some aspects of the recognition process. One potential interpretation is that there may be more than one way to recognize sick faces, either through a fast and automatic implicit process, used by the control group, or through a more controlled, explicit process, used by the disease training group. Impressively, both processes resulted in comparably accurate levels of sickness recognition accuracy.

The control group looked more at sick than healthy faces when making judgments about health. This result is in line with a previous study that found that faces edited to appear sick received more attention capture and holding compared to unedited healthy faces during a health-irrelevant facial categorization task [23]. Together, our findings and prior findings suggest that faces appearing sick may receive prioritized attention without priming or training, regardless of whether health is task-relevant, consistent with threat detection theories [21,22]. On the other hand, another study reported that healthy faces held attention longer than naturally sick faces in a passive viewing paradigm, possibly reflecting a bias to attend to faces of people who appear prosocial or attractive [19]. These different findings suggest that the instructions given to participants may influence how they naturally view faces varying in health.

In our study, we also found that the disease training group looked more equally at sick and healthy faces, potentially because they knew what they were looking for, leading them to more directly compare the two faces. In fact, compared to the control group, the disease training group looked more equally between the sick and healthy eye regions and showed marginally, though not statistically significantly, more alternating gaze shifts between the sick and healthy mouth regions. During the disease training, we highlighted changes in the eye and mouth regions of sick faces, because previous studies report that these are key features of sick faces [17,18]. Consistent with these visual attention results, the participants in the disease training group were more likely to report using the eye and mouth regions to determine which faces were sick, suggesting that they retained and applied the knowledge gained from the disease training. Overall, the participants’ visual attention and self-reports of the regions to which they attended reinforce the use of the eye and mouth regions in future sick face perception intervention studies.

## 5. Conclusions

Overall, our findings suggest that sick face perception may be malleable. The participants who engaged in training about disease were more accurate at avoiding sick people using only face photos compared to the participants in the control condition. Further, the disease training group looked more evenly between the sick and healthy faces than the control group, and they made more alternating gaze shifts between the sick and healthy mouths, in line with the disease training group being more likely to report using the mouth to decide which face was sick, a crucial piece of the lassitude expression [17]. This study supports the hypothesis that sick face perception is a plastic skill, flexibly influenced by experience, and serves as a first step to exploring ways to improve this ability. Future studies are still needed to develop ways to further strengthen sick face perception, test how long-lasting the effects are, and uncover the role of visual attention in driving this flexibility. Enhancing behavioral avoidance and visual attention to disease cues could reduce the spread of illness and maximize opportunities for social engagement.

## Figures and Tables

**Table 1:** Demographic information.

	Control Group	Disease Training Group
***Age in Years***		
Mean (SD)	19 (1)	19 (3)
Range	18–23	18–44
***Gender***		
Man	23	23
Nonbinary	1	1
Woman	42	43
***Ethnicity***		
Hispanic or Latino	18	12
Not Hispanic or Latino	46	54
Unknown or Other	5	2
***Race***		
American Indian or Alaska Native and White	0	1
Arab, Middle Eastern, or North African	1	1
Arab, Middle Eastern, or North African and Jewish	0	1
Arab, Middle Eastern, or North African and White	1	1
Asian	5	2
Asian and Native Hawaiian or Other Pacific Islander	1	0
Asian and Native Hawaiian or Other Pacific Islander and White	0	2
Asian and White	3	2
Black or African American	5	7
Black or African American and Native Hawaiian or Other Pacific Islander	1	0
Black or African American and White	2	1
Mixed (wrote-in)	0	1
Native Hawaiian or Other Pacific Islander	0	1
Unknown or prefer not to say	4	2
White	46	44
White and Unknown or prefer not to say	0	1

Note. Sample sizes for the control condition group (left) and disease training group (right).

**Table 2:** Summary of primary analyses and results.

Measure	Result
*Avoidance Task (Primary Analysis 1)*	
Accuracy	The disease training group was more accurate than the control group in the post-manipulation avoidance task; the disease training group was more accurate in the post-manipulation avoidance task than the pre-manipulation avoidance task.
Response latency	The disease training group was slower than the control group in the post-manipulation avoidance task; the control group was faster in the post-manipulation avoidance task than in the pre-manipulation avoidance task.
*Recognition Task (Primary Analysis 2)*	
Accuracy	n.s.
Look duration—Face	The disease training group showed more even looking at sick and healthy faces than the control group who looked more at the sick faces than the healthy faces.
Look duration—Eyes	n.s.
Look duration—Mouth	n.s.
Alternating gazes—Face	n.s.
Alternating gazes—Eyes	n.s.
Alternating gazes—Mouth	The disease training group showed marginally ^#^ more alternating gaze shifts than the control group.
*Self-Report (Primary Analysis 3)*	
Use of eyes	The disease training group was more likely to report using the eyes.
Use of mouth	The disease training group was more likely to report using the mouth.

Note: Accuracy measures were the number of correct trials. Duration-based measures (response latency and look duration) were difference scores (healthy minus sick) in seconds; n.s. indicates not statistically significant (*p*s > 0.05); ^#^ *p* = 0.062. Alternating gazes refer to the number of times the participants looked back and forth between the sick and healthy faces.

## Data Availability

Data associated with this paper are available in the Supplementary Materials.

## Supplementary Materials

The following supporting information can be downloaded at https://www.mdpi.com/article/10.3390/vision9020039/s1, supplementary materials and methods, Table S1, supplementary results, Table S2, avoidance task data, look duration data, alternating gaze shifts data.
