# Supplementary material for: Training Improves Avoidance of Natural Sick Faces: Changes in Visual Attention and Approach Decisions
Source: Vision (Basel). 2025 May 2;9(2):39. doi: 10.3390/vision9020039 (PMC12101381; doi:10.3390/vision9020039)
Supplement: Supplementary file 1 [file vision-09-00039-s001.zip › vision-3527268-supplementary/Supplementary Materials.pdf]

Supplementary Materials

Supplementary Materials and Methods

Face Stimuli

To confirm that donors were sick and healthy, we used validated, self-report health measures to capture symptoms, severity, and diagnoses (Andreasson et al., 2018; Arbuckle et al., 2021; CDC, 2021; Powell et al., 2008) at the time of each photo (see Table S1 for details). Face stimuli donors reported significantly better health (i.e., less severe sickness symptoms) at the time of donating their healthy face photo (“Healthy Faces” column) compared to the time they donated their sick face photo (“Sick Faces” column). Donors rated their symptom severities as None (0), Mild (1), Moderate (2), or Severe (3). Means (*M*) and standard deviations (*SD*) are reported for all available symptom scores.

Table S1. Healthy and Sick Face Stimuli: Symptoms and Severity

	Healthy Faces	Sick Faces				
	M (SD)	***M (SD)***	***t***	***df***	***p***	***d***
**Chills**	.00 (.00)	.75 (.93)	3.22	15	.006*	1.14
**Headache**	.13 (.34)	1.88 (1.20)	6.22	15	< .001*	1.98
**Joint pains**	.13 (.50)	.50 (.82)	2.09	15	.050*	.55
**Muscle or body aches**	.00 (.00)	1.47 (1.30)	4.36	14	< .001*	1.62
**Fatigue or tiredness**	.38 (.50)	2.31 (.87)	9.08	15	< .001*	2.72
**Nausea**	.00 (.00)	1.06 (1.18)	3.60	15	.003*	1.27
**Vomiting**	.00 (.00)	.38 (.89)	1.69	15	.111	.60
**Diarrhea**	.00 (.00)	.25 (.58)	1.73	15	.104	.61
**Abdominal pain**	.00 (.00)	.63 (.89)	2.82	15	.013*	1.00
**Sneezing**	.00 (.00)	1.00 (1.26)	3.16	15	.006*	1.12
**Sore throat**	.00 (.00)	1.88 (1.02)	7.32	15	< .001*	2.59
**Nasal discharge**	.00 (.00)	1.44 (1.03)	5.58	15	< .001*	1.97
**Nasal obstruction**	.00 (.00)	1.31 (1.20)	4.39	15	< .001*	1.55
**Cough**	.13 (.34)	1.25 (1.06)	4.14	15	< .001*	1.42

Note. Symptoms and severity reported by face photo donors. Degrees of freedom (*df*), and Cohen's *d* effect sizes are reported for each paired samples *t* test, *ps $\leq .05$.

Face photos were oval cropped around the face to ensure that effects would not be driven by external features (e.g., hairstyles. Within each sick-healthy face pair, we standardized face size using inter-pupil distance, and matched images on low-level features (e.g., luminance) using the SHINE_color toolbox (Dal Ben, 2021; Willenbockel et al., 2010). We assessed the low-level features of our stimuli (e.g., color, luminance, contour), using the Saliency Toolbox to

analyze each sick-healthy face pair (Ho-Phuoc et al., 2010; Walther & Koch, 2006). We tested whether the most salient regions were more likely to occur in the sick or healthy faces. We detected no difference in the proportion of trials in which the sick faces were the most salient compared to the proportion of trials in which the healthy faces were the most salient (.44 vs. .56, respectively), $\chi^2(1) = .50$, $p = .480$. These findings suggest that low-level features are unlikely to account for any potential differences between sick and healthy faces (Leung et al., 2023). Furthermore, given that the control group viewed the same face photo pairs as the disease training group, any group effects we observe cannot be attributed to low-level stimulus properties.

We drew oval areas of interest (AOIs) around each face that were the same size and shape within each face pair, but which varied slightly across pairs, to accommodate individual differences in the face stimuli. AOIs were sized 379-440 pixels wide ($M = 407.56$, $SD = 19.67$; range: 10.03-11.64 cm) × 515-744 pixels tall ($M = 589.44$, $SD = 53.86$; range: 13.63-19.68 cm), slightly extended over the edges of the stimuli by 50 pixels in each direction. Similarly, we drew rectangular AOIs around the eyes and mouths of each face that were the same size and shape within each face pair, but varied slightly in size and shape across pairs, to accommodate different face shapes. Eyes AOIs were sized 301-377 pixels wide ($M = 343.56$, $SD = 19.71$; range: 7.96-9.97 cm) × 89-121 pixels tall ($M = 101.56$, $SD = 7.50$; range: 2.35-3.20 cm), slightly extended over the edges of the stimuli. Mouth AOIs were sized 199-260 pixels wide ($M = 235.38$, $SD = 17.42$; range: 5.27-6.88 cm) × 96-128 pixels tall ($M = 110.13$, $SD = 9.37$; range: 2.54-3.39 cm), slightly extended over the edges of the stimuli (see Figure 1B for an example). We used the Velocity-Threshold Identification (I-VT) fixation filter in Tobii, which is commonly used and is robust to noise (Zeng et al., 2024). The I-VT fixation filter defines fixations by a velocity threshold of 30°/s, discards short fixations with a minimum duration of 100 ms, and merges adjacent fixations with a maximum time gap of 75 ms and a maximum angle of 0.5° (Olsen & Matos, 2012).

Supplementary Results

Data Inclusion

We excluded 3 participants (all in the disease training condition) for failing the attention check question (i.e., “Where did the story take place?”). For each measure, we checked for outliers (greater than or less than 1.5 times the interquartile range, calculated within each group). If there were outliers, we ran each analysis including the outliers and excluding the outliers, retaining all data when the results were unchanged, and removing the outliers when the results were different. We therefore excluded one participant (in the control group) from the pre-manipulation avoidance task, 3 participants (in the disease training group) from the recognition task for the alternating gaze shifts to the eye regions measure, and 3 participants (1 in the control group and 2 in the disease training group) from the recognition task for the alternating gaze shifts to the mouth regions measure. There were no other missing data.

Report of Facial Sickness Cues (Prediction 3)

Participants in the disease training group were more likely than those in the control group to report using the eyes and the mouth/lips to determine which person was sick. See Table S2 for representative examples from each group.

Table S2. Examples of Participants’ Descriptions of Facial Features of Sickness

Control group	Disease training group
If they weren't as healthy.	All the muscles are relaxed. It was a dead giveaway.
If they had chapped <u>lips</u> like they were dehydrated. They were sweatier and more tired looking.	Droopiness of the face, dark circles under the **eyes**, oily skin. Facial expression was agitated and unhappy.
Some look pale, exhausted, and sweaty.	The **eyes** looked like the life is drained out of their face.
They looked more pale, more sweaty, and drowsy.	They were sweaty and overheating. They had drooping **eyes** and <u>mouth</u>.

Sweat, general complexion, or if they looked too awake, nervous, or down.	Mostly the drooping **eyelids** and the relaxed face. They looked more tired.
Red cheeks and redness. They were sweaty, and tired.	**Eyes**, dark circles, paleness, skin tone and complexion. **Eyes** were watery or red. They had swelling and downturned corners of the lips.
Redness, pale, expressions.	Relaxed muscles, droopy **eyelids**, paleness sometimes, and a sad demeanor.
Faces looked distressed, upset, or tired. They had dark circles under their **eyes** and pale skin.	**Eyelids** drooping, downturned lips, puffy, red face. Their complexion just looks off.
They looked tired and less energetic. They had chapped lips.	If their **eyes** were droopy and the expression. When I'm sick, I'm dazed, so I was looking for lack of concentration or tiredness.
The **eyes** were tired, low, and sleepy. There was puffiness and redness.	They were more strained, like they were trying to stay awake. They had baggy **eyelids** and they were pale and puffy.
Facial expression. They looked tired, worried, less bright.	Facial muscles, the mouth, like the lips turned down, the **eyes** or **eyelids** were closed. They were tired. I just followed my gut.

Note. After participants completed the face recognition task (the last face perception task), the experimenter asked participants, “How did you decide which face was sick? Was there anything specific you were looking for to figure out if someone was sick?” Mentions of the eye region are in bold and mentions of the mouth/lip region are underlined.

Avoidance Task Data

- Columns C through R provide recoded values for each trial of the pre-manipulation avoidance task: 0 = incorrect (participant chose sick face), 1 = correct (participant chose healthy face)
- Columns S through AH provide manual response latencies (in seconds) for each trial of the pre-manipulation avoidance task
- Columns AI through AO provide responses for each of the 7 training trials during part 3 of the experimental manipulation
- Columns AP through BE provide recoded values for each trial of the post-manipulation avoidance task: 0 = incorrect (participant chose sick face), 1 = correct (participant chose healthy face)

- Columns BF through BU provide manual response latencies (in seconds) for each trial of the post-manipulation avoidance task
- Columns BV and BW provide the number of correct trials for the pre-manipulation and post-manipulation avoidance tasks
- Columns BX and BY provide the manual response latencies (in seconds), averaged across trials for the pre-manipulation and post-manipulation avoidance tasks

  Recognition Task Data
- Columns C through R provide recoded values for each trial of the recognition task: 0 = incorrect (participant chose healthy face), 1 = correct (participant chose sick face)
- Column S provides the number of correct trials for the recognition task
- Columns T through V provide values for whether the participant reported using the eye, nose, or mouth regions of the face: 0 = participant did not mention the face region, 1 = participant mentioned the face region

Look Duration Data

- Columns C through AH provide the total duration (in seconds) looking to the face AOIs
- Columns AI through BN provide the total duration (in seconds) looking to the eyes AOIs
- Columns BO through CT provide the total duration (in seconds) looking to the mouth AOIs
- Columns CU through DZ provide the total duration (in seconds) looking to the remaining face regions
- Columns EA through EP provide the look duration difference scores (sick face AOI - healthy face AOI) to the face AOIs
- Columns ER through FF provide the look duration difference scores (sick eyes AOI - healthy eyes AOI) to the eyes AOIs

- Columns FG through FV provide the look duration difference scores (sick mouth AOI - healthy mouth AOI) to the mouth AOIs
- Columns FW through GL provide the look duration difference scores (sick remaining face regions - healthy remaining face regions) to the remaining face regions
- Columns GN through GP provide the look duration difference scores to the face, eyes, mouth, and remaining face regions, averaged across trials

Alternating Gaze Shifts Data

- Columns C through CT provide the number of visits to each AOI. For example, column C "HealthyBoy1_Face" provides the number of visits to the face AOI for HealthyBoy1.
- Columns CU through EP provide the number of alternating gaze shifts for each face pair. For example, column CU "Boy1_Alternate_Face" provides the number of alternating gaze shifts between the SickBoy1 face AOI and the HealthyBoy1 face AOI.
- Columns EQ through ES provide the number of alternating gaze shifts the face, eyes, and mouth, averaged across trials.
